# Impact of Feature Selection Algorithm on Speech Emotion Recognition Using Deep Convolutional Neural Network

**DOI:** 10.3390/s20216008

**Published:** 2020-10-23

**Authors:** Misbah Farooq, Fawad Hussain, Naveed Khan Baloch, Fawad Riasat Raja, Heejung Yu, Yousaf Bin Zikria

**Affiliations:** 1 Department of Computer Engineering, University of Engineering and Technology, Taxila 47050, Pakistan; misbahfarooq222@gmail.com (M.F.); fawad.hussain@uettaxila.edu.pk (F.H.); naveed.khan@uettaxila.edu.pk (N.K.B.); 2 Department of Software Engineering, University of Engineering and Technology, Taxila 47050, Pakistan; faraja352@gmail.com; 3 Machine Intelligence and Pattern Analysis Laboratory, Griffith University, Nathan QLD 4111, Australia; 4 Department Electronics and Information Engineering, Korea University, Sejong 30019, Korea; 5 Department of Information and Communication Engineering, Yeungnam University, Gyeongsan 38541, Korea

**Keywords:** speech emotion recognition, deep convolutional neural network, correlation-based feature selection

## Abstract

Speech emotion recognition (SER) plays a significant role in human–machine interaction. Emotion recognition from speech and its precise classification is a challenging task because a machine is unable to understand its context. For an accurate emotion classification, emotionally relevant features must be extracted from the speech data. Traditionally, handcrafted features were used for emotional classification from speech signals; however, they are not efficient enough to accurately depict the emotional states of the speaker. In this study, the benefits of a deep convolutional neural network (DCNN) for SER are explored. For this purpose, a pretrained network is used to extract features from state-of-the-art speech emotional datasets. Subsequently, a correlation-based feature selection technique is applied to the extracted features to select the most appropriate and discriminative features for SER. For the classification of emotions, we utilize support vector machines, random forests, the k-nearest neighbors algorithm, and neural network classifiers. Experiments are performed for speaker-dependent and speaker-independent SER using four publicly available datasets: the Berlin Dataset of Emotional Speech (Emo-DB), Surrey Audio Visual Expressed Emotion (SAVEE), Interactive Emotional Dyadic Motion Capture (IEMOCAP), and the Ryerson Audio Visual Dataset of Emotional Speech and Song (RAVDESS). Our proposed method achieves an accuracy of 95.10% for Emo-DB, 82.10% for SAVEE, 83.80% for IEMOCAP, and 81.30% for RAVDESS, for speaker-dependent SER experiments. Moreover, our method yields the best results for speaker-independent SER with existing handcrafted features-based SER approaches.

## 1. Introduction

Speech is a natural and commonly used medium of interaction among human beings. The importance of speech in communication motivates many researchers to develop methods where speech can be used for human—machine interaction. However, the machine should be intelligent enough so that it can recognize not only speaker voices but also the emotional states of the speaker. In general, speech signals contain linguistic and paralinguistic information. Linguistic information refers to the language and context of the speech, whereas the paralinguistic information provides information about emotion in speech. In different parts of the world, people have different cultural backgrounds, local languages, speaking rates, and speaking styles. This cultural variation creates difficulties in the effective recognition of the emotional states of the speaker and makes the process of speech feature selection very challenging and complex. In the literature, acoustic features have been used by researchers for speech emotion recognition (SER) [1]. These acoustic features are further divided into four groups: continuous features (energy, pitch, formants, etc.), spectral features, qualitative features (voice quality), and Teager energy operator-based features. However, these handcrafted features mostly represent low-level features; therefore, they are not efficient for precise emotional classification in complex scenarios. Moreover, their performance degrades in complex situations, such as speaker and environment variations. Consequently, there is a need to extract the optimal and suitable features that are emotionally relevant by implementing efficient approaches for SER. For this purpose, we adopted a deep convolutional neural network (DCNN) that automatically extracts the relevant emotional features from the spectrogram of the speech signal. Several studies, such as [2,3], have been carried out in recent years, where a convolutional neural network (CNN) was implemented for feature extraction of speech. The 1-layer CNN was implemented in [2] for SER and, recently, an end-to-end SER system was implemented using a two-layer CNN followed by long short-term memory (LSTM) [3]. However, these 1-layer and 2-layer CNNs are not suitable for learning emotional discriminative features due to their shallow architectures. In [4], DCNNs, which consist of deep multilevel convolutional layers and pooling layers, were adopted. Because DCNNs involve more parameters to extract more detailed temporal frequency correlation information and have strong feature learning ability, they achieve better performance than shallow CNNs.

In this study, deep features are extracted from a DCNN, and a correlation-based feature selection (CFS) technique is applied to select the most discriminative features for SER. We use support vector machines (SVMs), random forests (RFs), the K-nearest neighbors (KNN) algorithm, and multilayer perceptron (MLP) classifiers for emotion recognition. For performance assessment, we use four publicly available datasets: the Berlin Dataset of Emotional Speech (Emo-DB) [5], Surrey Audio Visual Expressed Emotion (SAVEE) [6], Interactive Emotional Dyadic Motion Capture (IEMOCAP) [7], and the Ryerson Audio Visual Dataset of Emotional Speech and Song (RAVDESS) [8]. Due to many factors, including cultural background, accent, and gender, the expression of emotion varies among various speakers. The proposed strategy provides better accuracy for both speaker-dependent and speaker-independent scenarios. The main contributions of this study are as follows;

1. An algorithm using a DCNN to extract emotional features for SER is proposed.
2. A CFS algorithm, which leads to improved accuracy for SER, is used.
3. The proposed method achieves performance improvement over the existing handcrafted and deep learning SER approaches for both speaker-dependent and speaker-independent scenarios.

The remainder of this paper is structured as follows. In Section 2, the various existing methods used in the literature for SER are discussed. Section 3 provides the details of the proposed methodology. Section 4 presents details of the benchmark datasets for SER. In Section 5, we provide the experimental results and discussion. In Section 6, the conclusions are given, and topics for future research are discussed.

## 2. Literature Review

There are two primary components of SER. The first component is discriminative feature extraction from speech signals, and the second is the selection of the classifier that categorizes the emotion from speech utterances. We briefly discuss the classification strategies and feature extraction methods related to SER.

### 2.1. Emotion Classifier

There are several machine learning-based classifiers that have been used by researchers to distinguish emotional classes: SVMs [9], RFs [10], the KNN algorithm [11], hidden Markov models (HMMs) [12], MLPs [13], and Gaussian mixture models (GMMs) [14]. These classifiers have been widely used for speech-related tasks. In this study, SVM, RF, MLP, and KNN classifiers are used as benchmarks. We evaluate the performance of these classifiers in terms of accuracy.

### 2.2. Feature Extraction

Two approaches have been used in the literature for feature extraction. One commonly used approach is to split the speech signal into short intervals, called frames, and extract local features from each frame. In [14,15], prosodic features (pitch and energy) were extracted from each speech interval. In the second approach, global features are extracted from the entire speech signal [16,17,18]. In [17], 98 pitch-based features were used for the classification of seven emotions from the Emo-DB dataset. In general, there are four categories of speech features that are used for SER: acoustic features, context information, linguistic features, and hybrid features (that contain information of acoustic features with other statistics). Acoustic features are the most popular and widely used for SER. They include prosodic features (pitch, loudness, and duration), voice quality features, and spectral features [19]. The voice quality features are the harmonics-to-noise ratio, spectral energy distribution, the first three formants, jitter, and shimmer. The most widely used spectral features are mel-frequency cepstral coefficients (MFCCs), linear prediction cepstral coefficients (LPCC), and log frequency power coefficients. In [20], the fundamental frequency, formants, energy, and mel sub-band energy features were extracted from speech data. In the next step, an HMM was used to classify the emotions. In [21], handcrafted features, such as voice quality and prosody features, were extracted for SER. Evaluations were performed on a Chinese natural emotional speech dataset to classify four emotional classes: angry, joy, sad, and neutral. Some speech features are based on context information. The context information conveys the information of the speaker and spoken content. In [22,23], context information was used for effective emotion recognition. In [23], speaker information, spoken content, and gender information were extracted from Emo-DB and a locally collected audiovisual database in a car setting (CVRRCar-AVDB) for SER. The linguistic information (content of a spoken utterance) also plays an important role in detecting emotions from speech. A spotting algorithm that examines spoken utterances for emotional keywords or phrases was proposed in [9]. The keyword spotting algorithm deals with the detection of keywords in the utterances. In [24], lexical information was used for detecting emotions from speech signals. A feature extraction technique based on a bag-of-audio-words representation of MFCCs was proposed in [25]. Evaluation is performed using the RECOLA dataset [26]. Support vector regression was used to predict emotions in the arousal and valence dimensions. Another feature set, proposed by the INTERSPEECH emotion challenge and paralinguistic challenges in [27,28,29,30], was used for effective SER. In [31], an ensemble softmax regression model was proposed for SER. Spectral and prosodic features were extracted from two datasets, Emo-DB and SAVEE. MFCC, LPCC, perceptual linear prediction, and RASTA perceptual linear prediction are the spectral features. The INTERSPEECH 2010 (IS10) feature set was obtained using the OpenSMILE toolkit, which represents the prosodic features.

Recently, it has been shown that deep learning systems, such as DCNNs, have the ability to learn emotional features for SER. Multitask learning based on a CNN was implemented in [32] for SER. It involves arousal level, valence level, and gender-based classification. Consequently, it can reduce the generalizing error problem and provide higher accuracy. In [33], a frame-level hybrid deep belief network (DBN) followed by an HMM classifier was proposed with the FAU Aibo dataset [19] to classify the five emotion classes. In [34], an automatic SER system using a recurrent neural network (RNN) with local attention was proposed. A bidirectional LSTM with a pooling strategy, which focuses on emotionally relevant parts of utterances, was used. Evaluation was performed using the IEMOCAP dataset. Another efficient technique used for emotion recognition from speech signals based on convolutional LSTM was proposed in [35]. The convolutional networks are capable of learning spatial patterns and effectively learn spatial spectrogram patterns that depict emotional state information. A multitask model based on gated residual networks was used for audio classification [36]. The purpose of using this network is to recognize emotional states from speech and song data. It can also be used for accents and speaker recognition. In [37], an adversarial data augmentation network (ADAN) was proposed to produce synthetic samples for data augmentation to solve the problem of data insufficiency. Evaluation was performed on the EmoDB and IEMOCAP datasets using openSMILE features as inputs, where SVM and a deep neural network were used as classifiers for emotion classification. Multitask learning to extract the activation and valence information for audio emotion recognition based on the DBN framework was proposed in [38]. Evaluation was performed on the IEMOCAP dataset to classify the four emotional states.

In [39], features were extracted from the IEMOCAP dataset by integrated attention-based bidirectional LSTM (BiLSTM) RNNs with fully convolutional networks in order to automatically learn the best spatio-temporal representations of speech signals. Another technique based on combining amplitude and phase information using a CNN was studied in [40] for SER. Unsupervised learning of unlabeled speech was proposed in [41] for SER. The auDeeptoolkit [42] was employed to extract the spectrogram, train the autoencoder, and generate the representation for a model. A deep spectrum feature representation from a spectrogram by an attention-based LSTM with fully convolutional networks was proposed in [43]. DeepSpectrum is a Python-based toolkit for extraction of features from audio signals using a pretrained CNN. Evaluation was performed on IEMOCAP for four emotion classifications: angry, happy, sad, and neutral. In [44], a CNN was employed for the recognition of four emotional states, happy, neutral, angry, and sad, from the IEMOCAP dataset. In [45], a combined architecture with a CNN and LSTM with data augmentation was proposed. Evaluation was performed on IEMOCAP to classify the four emotion classes. In [46], a 3-D convolutional RNN with an attention model was proposed for SER. Evaluation was performed on two datasets, Emo-DB and IEMOCAP, for classification of emotion. A Clustering-based speech emotion recognition was proposed in [47] by using Resnet model and deep BiLSTM. Evaluation was performed on the three datasets for the classification of emotions. In [48] an exploration of complementary features based on the kernel extreme learning machine (KELM) was proposed for SER. Experiments were conducted on Emo-DB and IEMOCAP dataset for the recognition of emotional classes. In [49], a dilated CNN with a residual block and a BiLSTM built on the attention mechanism architecture was proposed. The local correlations and global contextual statistics were investigated from 3-D log-mel spectrograms of speech signals for recognition of speech emotion. However, these deep learning approaches require a large amount of training data and have a high computational cost. Most existing speech emotional datasets have a limited amount of data. They are not sufficient to train deep learning models with a large number of parameters. Based on the above analysis, a pretrained network, AlexNet, was used for efficient emotional feature extraction. A transfer learning technique using a pretrained DCNN was proposed in [50] for SER. Improved results were achieved for seven emotion classes. In [51], a transfer learning-based approach was employed for improving the classification accuracy in cross-language and cross-corpus experiments. Evaluation was performed using five datasets in three different languages. It showed superior accuracy compared with existing approaches. A DCNN followed by discriminant temporal pyramid matching was proposed in [52]. The AlexNet model was used for feature extraction from its fully connected layer (FC7). Evaluation was performed on four datasets: EMO-DB, RML [53], eNTERFACE05, and BAUM-1s [54]. An SVM classifier was used for the classification of emotion. The main advantages of using pretrained networks are to save training time and provide better performance of the neural network, as well as the fact that they require less training data, and directly deal with the complex factors of variations.

## 3. Methodology

**Figure 1:**
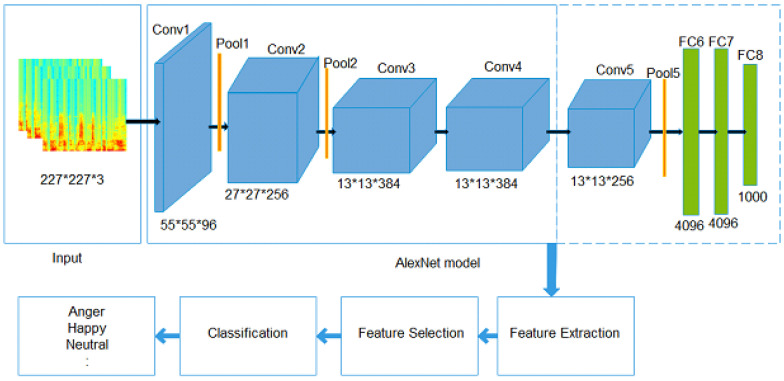
The framework of our proposed methodology.

This section describes the proposed algorithm using a pretrained DCNN, AlexNet, for SER, as shown in Figure 1. The AlexNet model [4] is pretrained on the large-scale ImageNet database, which represents a large number of different object classes and consumes less training time to adapt the structure for a new classification problem. The AlexNet model is also used for SER. The structure of the pretrained AlexNet is comprised of an input layer, convolutional layers, pooling layers, and fully connected layers (FCLs). In the proposed work, the convolutional layer (Conv4) of the model is investigated to obtain the lower-level representation. The speech signals are converted into spectrograms, which are computed by applying the fast Fourier transform (FFT) to emotional speech signals. The spectrograms show the time–frequency representations of the signals. The spectrogram has been extensively used in speaker and gender recognition [55]. In this paper, we explore their effects on SER in terms of recognition accuracy. The proposed methodology is described in the following subsections.

### 3.1. Speech Emotion Recognition Using a Deep CNN

The architecture of a DCNN is shown in Figure 1. The features are extracted from the convolution layer (Conv4) of the pretrained network, which is followed by a feature selection technique to select the most discriminative features. We use a CFS technique that selects discriminative features. The CFS technique evaluates the subset of attributes and selects only those features that have a high correlation with the output class label. The classification algorithm is used on these features to assess the performance in terms of accuracy. A brief description of each step is given in the following subsections.

#### Features Extraction

Feature extraction is performed using AlexNet, a pretrained DCNN. In AlexNet, the original weights of the network remain fixed, and the original layers are used for the feature extraction process. AlexNet has a deep architecture with more filters per layer and has stacked convolutional layers. It contains convolutional layers, max-pooling layers, dropout, rectified linear unit (ReLU) activations, data augmentation, and stochastic gradient descent with momentum. The AlexNet model uses ReLU activation, which accelerates the training process. The DCNN layers are briefly described as follows.

(a) **Input layer**
  The first layer is the input layer. The AlexNet model uses an input with a size of 277×227×3. Therefore, we resized the spectrogram of the speech signal into a compatible size of input layer.
(b) **Convolutional Layer (CL)**
  The CL is comprised of convolutional filters that are used to extract several local patterns from every local area in the input, and generates several feature maps. There are five convolutional layers, Conv1, Conv2, Conv3, Conv4, and Conv5, in the AlexNet model, and three of them (Conv1, Conv2, and Conv5) are followed by max-pooling. The convolutional layers use the ReLU activation function. The first convolutional layer (Conv1) has 96 kernels of size 11×11×3 with a stride of 4 pixels and zero-padding. The second (Conv2) has 256 kernels of size 5×5×48 with a stride of 1 and a padding value of 2. The third convolutional layer (Conv3) has 384 kernels of size 3×3×256 connected to the outputs of Conv2, and the fourth convolutional layer (Conv4) has 384 kernels of size 3×3×192. The ReLU activation function is adopted at the output of every convolutional layer, which accelerates the training process.
(c) **Pooling Layer (CL)**
  The PL is employed after the convolutional layers. The purpose of the PL is to down-sample the feature maps obtained from the preceding convolutional layers to generate a single output convolutional feature map from the local regions. There are two general pooling operators: max-pooling and average pooling. The max-pooling layer generates a reduced resolution form of convolutional layer activations by utilizing maximum filter activation from distinct positions within a quantified window.
(d) **Fully Connected Layers (FCLs)**
  This layer combines the features obtained from the preceding layers and produces a feature representation for the classification task. The outcome from the convolutional and pooling layers is given to the FCL. In the AlexNet model, there are three FCLs: FC6, FC7, and FC8. FC6 and FC7 produce a 4096-dimensional feature vector, whereas FC8 yields a 1000-dimensional feature vector.

Fully connected Layers can be used to extract feature representations. These are the universal approximators; however, FCLs do not perform well at identifying and generalizing the raw image pixels. Whereas Convolutional Layers (Conv4) extract meaningful features from raw pixel values by preserving spatial relations within the image. FCL extracts global features, whereas Convolutional Layers (CLs) generate local features and make local descriptors into a compact feature vector. Therefore, in the proposed scheme, features are extracted from the convolutional layer (Conv4) that are used for speech emotion recognition. The local connectivity of the convolutional layer (Conv4) allows the network to learn filters that respond maximally to a local input region, thus leveraging the spatial local correlation of the input. A total of 64,896 features were obtained from the Convolutional Layer (Conv4). These extracted features are followed by a feature selection technique and pass through a classifier for classification.

### 3.2. Feature Selection

This procedure determines the discriminative and relevant features for model development. Feature selection techniques are deployed with simple models so that they take less training time and improve the generalization capability by reducing overfitting. Its primary purpose is to remove the features that are redundant and insignificant.

#### Correlation-Based Feature Selection (CFS) Method

A CFS method [56] is employed in this study. The CFS method evaluates the subset of attributes and selects only the discriminative features that have a high correlation with a class instance. CFS ranks the attributes using a heuristic evaluation function based on correlation. It is used to measure the similarity between features. CFS discards irrelevant features that have less correlation with the class label. The CFS criterion is as follows:

(1) $$CFS=max\left[\frac{r_{cf1}+r_{cf2}+..+r_{cfk}}{\sqrt{k+2(r_{f_{1}f_{2}}+..+r_{f_{i}f_{j}}+..+r_{f_{k}f_{k-1}}})}\right]$$

where $r_{cfi}$ is a feature classification correlation, *k* is the number of features, and $r_{f_{i}f_{j}}$ represents the correlation between features. The chosen features are provided to the classifiers for SER.

### 3.3. Classification Algorithms

The discriminative features are given to the classifiers for an emotion classification task. In this study, four different classifiers, MLP, SVM, KNN, and RF, are used to evaluate their performance for SER. Each classifier is briefly described in the following subsections.

#### Support Vector Machine (SVM)

The SVM is a nonlinear supervised learning algorithm adopted for binary classification or regression. It constructs an optimal hyperplane in which the margin between the classes is maximized. The support vectors indicate a small subset of training data that are used to define the hyperplane. SVMs need to recognize the support vectors $c_{i}$, weights $w_{gi}$, and bias *b* to classify the input data. The following equation is used to classify the data:

(2) $$k\left(c,c_{i}\right)={\left(\gamma c^{t}c_{i}+m\right)}^{d}$$

where *m* is a constant and *d* represents the degree of the polynomial. For polynomial functions γ>0:

(3) $$c=\sum_{i=1}^{n}w_{gi}k\left(c_{i},c\right)+b$$

where *k* is a kernel function, *c* represents input data, $c_{i}$ is a support vector, $w_{gi}$ represents a weight, and *b* is a bias. The polynomial kernel is used in our experiment, which maps input data into a higher-dimensional space.

### 3.4. Random Forests

An RF is an ensemble learning classifier that is mainly used for classification and regression. For the training of data, it forms a multitude of decision trees, which gives the output of the class, and is a mean indicator of the individual trees. An RF randomly samples each individual tree from the database with a replacement, which results in distinct trees. This procedure is called bagging. In the RF, classifier node splitting is based on an arbitrary subset of features for each tree.

### 3.5. k-Nearest Neighbors Algorithm

The KNN method stores all instances of data. Based on a similarity measure, it finds the *K* most similar training instances and applies the majority class emotion to these *K* instances. In this study, we set K=10 for emotion recognition. The KNN algorithm uses the Euclidean distance to find ten nearest neighbors, and emotion classification is performed on the basis of a majority vote.

### 3.6. Multilayer Perceptron

MLPs are a commonly used feedforward artificial neural network. They are comprised of several layers of computational units. MLPs can solve classification problems. They utilize a supervised backpropagation learning technique for the classification of instances. The MLP classifier is comprised of an input layer, hidden layers, and an output layer. The input layer has neurons, of which the number is equal to the number of features. The number of hidden layers is determined by the average number of emotions in the dataset and features dimensionality after CFS. The number of output neurons is equal to that of the emotions(classes) in the dataset. In this study, the sigmoid function is used as an activation function, as follows:

(4) $$y_{i}=\frac{1}{1+e^{-x_{i}}},$$

where $y_{i}$ represents the state and $x_{i}$ is the total weighted input. There is one hidden layer in the MLP for the Emo-DB dataset and it contains 233 neurons, whereas with the SAVEE dataset it includes one hidden layer that contains 80 neurons. For the IEMOCAP dataset, there is single hidden layer and 138 neurons, while MLP contains one hidden layer and 275 neurons for the RAVDESS dataset. MLP is a two-level framework which means that the classification task of MLP is a two-stage process—i.e., training and testing. In the training process, the weight values are determined to match them to the specific output class.

## 4. Experiments

### 4.1. Datasets

The proposed approach is evaluated using four databases, as follows:

- **Emo-DB:** The Emo-DB speech corpus consists of seven acted emotional states: angry, disgust, boredom, joy, sad, neutral, and fear. It has 535 emotional utterances in the German language collected from ten native German actors. Among them, five are female actors and the remaining five are male actors. The audio files have a sampling frequency of 16 kHz and have 16-bit resolution. The average time period of the audio files is 3 s.
- **SAVEE:** The SAVEE dataset was recorded with a high-quality audio–visual apparatus in a visual media laboratory. It has 480 British English emotional utterances recorded from four male actors. It consists of seven emotional states: anger, frustration, happiness, disgust, neutral, surprise, and sadness. The audio files had a sampling frequency of 44.1 kHz, and the recorded data were evaluated by ten subjects.
- **IEMOCAP:** This is an acted and multi-speaker dataset that was collected at the Signal Analysis and Interpretation Laboratory at the University of Southern California. The dataset consists of five sessions, and in each session, two English-speaking actors (one male and one female) are engaged in scripted or improvized scenarios to elicit desired emotions. The recorded audio has a sampling frequency of 16 kHz. Multiple professional annotators have annotated the IEMOCAP database into categorical labels. Each utterance was assessed by three different annotators. We assigned labels to the utterances on which at least two annotators agreed. In this study, four emotional classes are employed: happiness, sadness, neutral, and angry.
- **RAVDESS:** RAVDESS is an audio and visual emotion dataset that contains eight emotional states: angry, neutral, calm, happy, sadness, fear, disgust, and surprise. The emotional utterances were recorded in a North American accent from 24 professional actors, in which 12 are female actors and 12 are male actors. The audio files have a sampling frequency of 48 kHz with 16-bit resolution.

### 4.2. Experimental Setup

#### 4.2.1. Data Pre-Processing

The pre-processing consists of a data-mining process for the conversion of data into a specific format. We converted the speech signal into a log-mel spectrogram representation. Because AlexNet requires an input layer of size 227×227×3, the spectrograms are resized according to the input layer size. Afterwards, a spectrogram representing audio data is fed into a pretraining network for the feature extraction process. Experiments were performed on a MATLAB 2018 platform [57] with an Intel Core i7 processor with 8 GB of RAM and a 64-bit OS. The Deep Learning Toolbox and the AlexNet Network support package are also used in the system.

#### 4.2.2. Evaluation Parameters

In this study, speaker-dependent and speaker-independent SER experiments are performed on four benchmarked datasets. We used the weighted average recall (WAR) to evaluate the accuracy of the proposed methodology. WAR calculates the number of correctly predicted samples in the class divided by the total number of samples in that class. Because the datasets have imbalanced classes, we applied a data resampling technique to achieve a better training process and results. Data resampling can be performed in two ways: oversampling and undersampling. Oversampling increases the number of samples in the minority class, whereas in undersampling, the samples in the larger class are reduced by removing the samples until the dataset becomes balanced. In our study, we used a supervised resampling filter to oversample the minority class [58].

## 5. Results Analysis and Discussion

### 5.1. Speaker-Dependent Experiments

We assessed the proposed approach using benchmarked datasets for speaker-dependent experiments. We applied a ten-fold cross-validation technique to our evaluations. The data are randomly split into 10 equal parts for training and testing processes with a splitting ratio of 90:10. Table 1 represents the results obtained from the four classifiers using the features obtained from convolutional layer 4 of AlexNet (Conv4). With the Emo-DB dataset, the highest accuracy of 91.11% was obtained through SVM. The SVM achieved an accuracy of 79.08% with the SAVEE database, while the MLP achieved an accuracy of 80.00% with the IEMOCAP set. The SVM achieved 80.97% accuracy with the RAVDESS dataset.

Table 2 shows the results of feature extraction followed by the feature selection and data resampling technique, which selects the most discriminative features and then applies classification algorithms for emotion classification. The CFS technique obtained the 458 most discriminative features out of 64,896 features for Emo-DB, whereas the CFS technique selected 150 out of 64,896 features for the SAVEE dataset. Moreover, the CFS technique obtained 445 and 267 features with the IEMOCAP and RAVDESS datasets, respectively.

The experimental results show that there is a significant improvement in accuracy by using feature selection and a data resampling strategy. We report the weighted average recall and standard deviation from ten-fold cross-validation to assess the stability and performance of speaker-dependent experiments using a feature selection technique. The best performance of the DCNN followed by the feature selection technique reached 95.10% with the Emo-DB dataset through SVM. The SVM achieved 82.10% accuracy for SAVEE, while the MLP achieved 83.80% accuracy for IEMOCAP. The SVM obtained an accuracy of 81.30% with the RAVDESS dataset.

**Figure 2:**
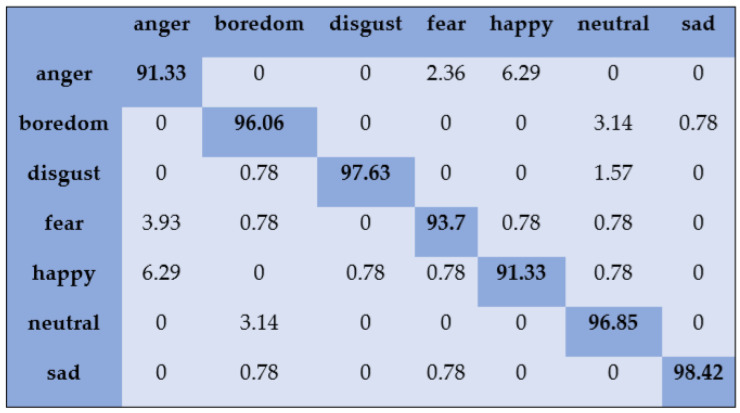
Confusion matrix on Emo-DB dataset for speaker-dependent SER.

To analyze the recognition accuracy of the individual emotional classes, the confusion matrix of the results is utilized in this study. With the Emo-DB dataset, “disgust” and “sad” are recognized with higher accuracy with the SVM classifier with feature selection compared to the other emotional classes, as shown in Figure 2.

**Figure 3:**
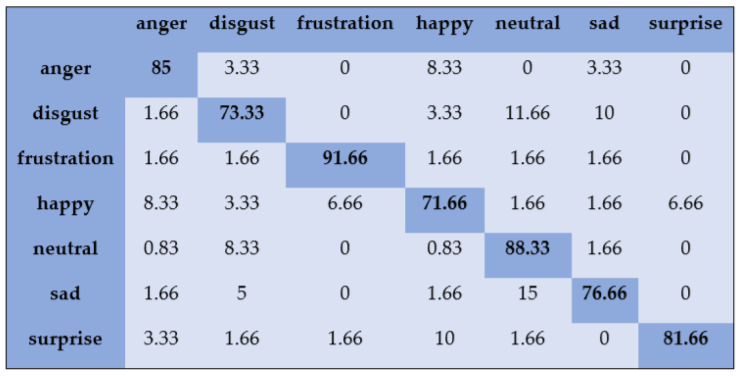
Confusion matrix of SAVEE dataset for speaker-dependent SER.

**Figure 4:**
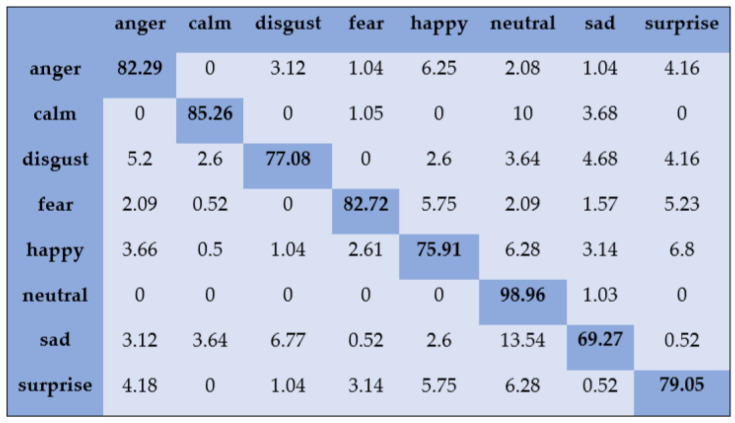
Confusion matrix of RAVDESS dataset for speaker-dependent SER.

**Figure 5:**
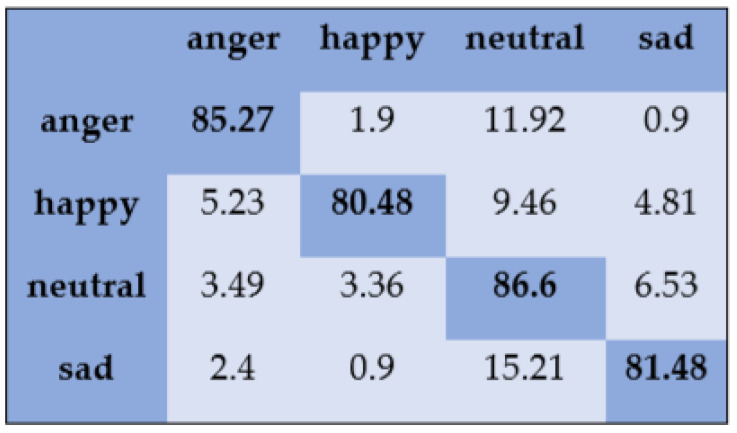
Confusion matrix of IEMOCAP dataset for speaker-dependent SER.

With the SAVEE dataset, “neutral” and “frustration” were identified with higher accuracies of 88.33% and 91.66% through SVM, as shown in Figure 3. There are eight emotions in the RAVDESS dataset, where “calm” and “neutral” are classified with accuracies of 85.26% and 98.96%, respectively, as shown in Figure 4. With the IEMOCAP dataset, “neutral” was identified with a high accuracy of 86.20%, whereas “happy,” “anger,” and “sad” were classified with accuracies of 80.48%, 85.27%, and 81.48%, respectively, as shown in Figure 5.

### 5.2. Speaker-Independent Experiments

**Figure 6:**
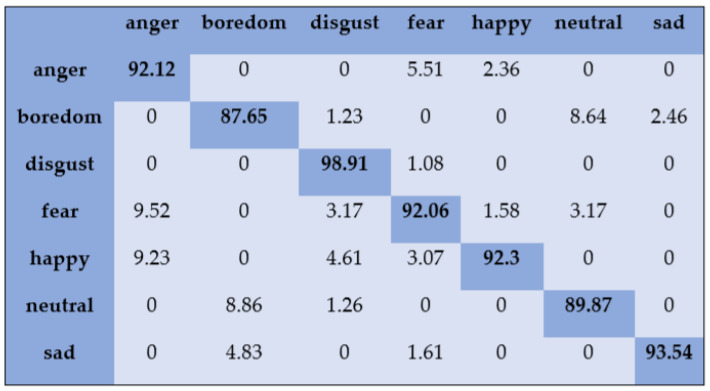
Confusion matrix of Emo-DB dataset for speaker-independent SER.

**Figure 7:**
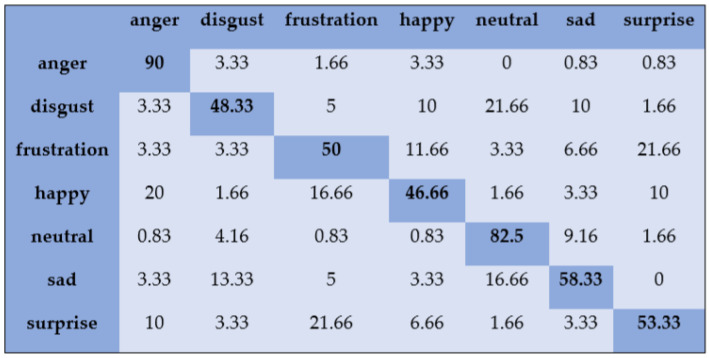
Confusion matrix of- SAVEE dataset for speaker-independent SER.

**Figure 8:**
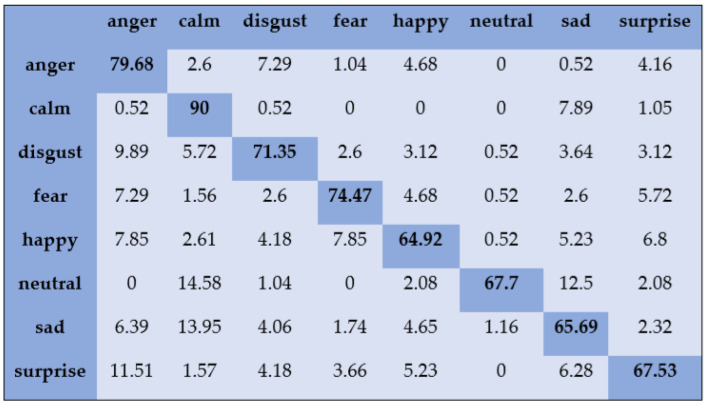
Confusion matrix of RAVDESS dataset for speaker-independent SER.

**Figure 9:**
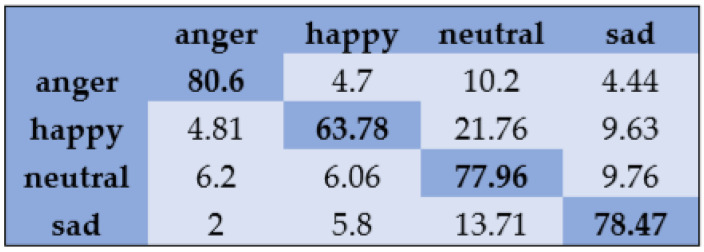
Confusion matrix of IEMOCAP dataset for speaker-independent SER.

We employed the leave-one-speaker out (LOSO) scheme for speaker-independent SER experiments, in which one speaker is selected for testing and the rest of the speakers are used for training purposes. For the IEMOCAP database, one session was used for testing, while the remaining sessions were used for training purposes. The process is repeated by reversing all the testing speakers. The average accuracy was obtained from all the testing speakers. The results obtained by the four classification algorithms for speaker-independent experiments without feature selection are shown in Table 3. The results show that the highest accuracy of 82.00% is achieved by the neural network with the Emo-DB dataset. The KNN achieved an accuracy of 58% with the SAVEE database, while the RF achieved an accuracy of 56.51% with IEMOCAP, and SVM obtained a 63% accuracy with RAVDESS. Table 4 shows the results of feature extraction followed by CFS and the data resampling technique for speaker-independent SER experiments. The experimental results show that the feature selection and data resampling strategy improved the accuracy. We report the weighted average recall and standard deviation to assess the stability and performance of a speaker-independent experiment using the feature selection technique. The best performance of the DCNN followed by the feature selection technique for speaker-independent experiments reaches 90.50% for the Emo-DB dataset, 66.90% with SAVEE, 76.60% with IEMOCAP, and 73.50% with the RAVDESS dataset. To analyze the recognition accuracy of the individual emotional classes, we show the confusion matrices of the obtained results for speaker-independent SER experiments in Figure 6, Figure 7, Figure 8 and Figure 9.

With the Emo-DB dataset, “anger,” “disgust,” “fear,” “happy,” and “sad” are recognized with an accuracy higher than 90% by the MLP classifier with feature selection, as shown in Figure 6. The average accuracy obtained with the Emo-DB dataset is 90.50%. For the SAVEE dataset with seven emotion classes, “anger” and “neutral” are classified with accuracies of 90.00% and 82.50%, respectively, by the MLP classifier, whereas the other four emotions are recognized with accuracies below 60.00%, as shown in Figure 7. The average accuracy obtained with the SAVEE dataset is 66.90%. With the RAVDESS dataset, “anger,” “calm,” and “fear” are classified with higher accuracy by the MLP classifier, as shown in Figure 8. The average accuracy obtained with the RAVDESS dataset is 73.50%. Figure 9 indicates that “anger,” “neutral,” and “sad” are distinguished with accuracies higher than 77% with the IEMOCAP dataset, whereas the “happy” emotion is identified with an accuracy of 63.78%. The average accuracy obtained with the IEMOCAP dataset is 76.60%.

### 5.3. Comparison with State-of-the-Art Approaches

In this section, a comparison of the proposed work is performed with four state-of-the-art datasets. As shown in Table 5, the proposed method achieved better results than [40,46,49,59,60] with the Emo-DB dataset for speaker-dependent SER. In [59], features were extracted using OpenSMILE software for SER. They achieved accuracies of 84.62% and 72.39% with the Emo-DB and SAVEE datasets, respectively. These handcrafted features were extracted manually from the dataset. For the IEMOCAP dataset, the proposed technique achieved promising performance compared to [35,39,45,46,49]. The authors constructed a 3-D convolutional RNN with an attention model (ACRNN) in [46]. A combined CNN and LSTM architecture with data augmentation was proposed in [45]. In [39], a dilated CNN with a residual block and a BiLSTM built on the attention mechanism architecture (ADRNN) was proposed for SER. However, the architecture requires a long training time and is computationally complex. In our approach, features are extracted from a pretrained DCNN, and we then apply the CFS technique. This reduces a significant amount of the workload for the classifiers and improves the performance. With the RAVDESS dataset, the proposed method improves the results in terms of accuracy compared to [36,60].

Table 6 indicates that the proposed methodology achieves better performance compared to [31,37,47,48,49,52,61] with the Emo-DB dataset for the speaker-independent experiments. The authors in [31,37,61] extract low-level descriptor acoustic features for SER. They achieved 82.40%, 76.90%, and 83.74% accuracy with the Emo-DB dataset. Deep learning approaches were also used for emotion recognition in [47,48,49,52] with Emo-DB. The SAVEE dataset is a relatively small dataset compared with other speech emotion datasets. The main advantage of using a pretrained DCNN is that it can easily be trained with a small dataset. With SAVEE, the proposed method gives the best results in terms of accuracy compared with [31,61]. With the IEMOCAP dataset, the proposed technique shows better accuracy than [37,38,44,47,48,49]. According to the classification results, there is a significant improvement in accuracy over the existing approaches. For instance, the proposed scheme achieves 73.50% accuracy with the RAVDESS dataset.

## 6. Conclusions and Future Work

In this study, the main focus was on learning relevant and discriminative features from state-of-the-art speech emotional datasets, which is a critical research problem for SER. In this regard, the major contribution of the proposed work is automatic feature learning using a DCNN. We proposed a CFS algorithm that examines the predictive capability of each feature, reduces the feature-to-feature correlation, and increases the feature-to-output correlation. The experimental results with four benchmarked speech emotional datasets verify the superior performance of the proposed method for both speaker-dependent and speaker-independent scenarios. The CFS technique obtained the 458 most discriminative features out of 64,896 features with Emo-DB. It achieved 95.10% accuracy for the SVM for speaker-dependent experiments and 90.50% accuracy for the MLP classifier for speaker-independent experiments. However, with the SAVEE dataset, the CFS technique selected 150 out of 64,896 features and obtained 82.10% accuracy for the SVM for speaker-dependent experiments, and 66.90% accuracy for the MLP classifier for speaker-independent experiments. With the IEMOCAP dataset, the CFS technique achieved the best accuracy, 83.80%, for the MLP classifier for speaker-dependent experiments, and 76.60% accuracy for the SVM classifier for speaker-independent experiments using 445 features. In contrast, with the RAVDESS dataset, the CFS technique yielded the best accuracy, 81.30%, for the SVM classifier, and 73.50% for the MLP classifier for speaker-dependent and speaker-independent experiments by using only 267 features. To analyze the recognition accuracy of the individual emotional classes, we presented the confusion matrices of the obtained results. The advantage of using the feature selection technique is the reduction in the number of features by choosing the most discriminative features and discarding the remaining less-effective features. By doing so, the workload of the classifiers was dramatically reduced. In addition, we found that pretrained DCNN models are very effective for speech emotion feature extraction, and can be easily trained with a limited labeled speech emotional database. The success of our work motivates us to explore the effectiveness of this approach for analyzing the effect of gender on speech emotion signals, and also for cross-language datasets for effective emotion recognition. We will conduct the training and testing procedures using the datasets of different languages, which should be an interesting test of our proposed method. 

## Figures and Tables

**Table 1:** Weighted average recall and standard deviation of Speaker-dependent SER experiments without feature selection.

Dataset	MLP	SVM	RF	KNN
Emo-DB	90.10±2.61	91.11±2.58	76.04±3.23	81.55±3.51
SAVEE	79.01±4.50	79.08±4.59	73.33±5.82	74.16±5.18
IEMOCAP	80.00±3.78	73.19±3.91	75.77±3.82	67.59±4.92
RAVDESS	76.00±3.36	80.97±3.66	73.51±4.21	73.37±4.02

**Table 2:** Weighted average recall and standard deviation of speaker-dependent SER experiments with feature selection.

Dataset	MLP	SVM	RF	KNN
Emo-DB	94.12±2.31	95.10±2.25	93.70±2.68	90.80±2.59
SAVEE	81.50±4.20	82.10±4.38	81.70±4.52	78.31±4.98
IEMOCAP	83.80±3.14	69.10±4.47	81.20±3.08	76.66±3.20
RAVDESS	79.20±3.24	81.30±3.21	80.60±3.68	81.10±3.95

**Table 3:** Weighted average recall and standard deviation of speaker-independent SER experiments without feature selection.

Dataset	MLP	SVM	RF	KNN
Emo-DB	82.00±3.02	80.37±3.16	74.92±4.78	70.00±4.92
SAVEE	55.00±6.74	52.91±6.18	47.49±7.21	58.00±6.34
IEMOCAP	52.00±5.92	51.86±6.97	56.51±5.23	44.47±6.27
RAVDESS	60.71±4.95	63.00±4.28	56.50±5.97	45.00±6.02

**Table 4:** Weighted average recall and standard deviation of speaker-independent SER experiments with feature selection.

Dataset	MLP	SVM	RF	KNN
Emo-DB	90.50 ± 2.60	85.00 ± 2.95	80.15 ± 2.68	78.90 ± 2.92
SAVEE	66.90 ± 5.18	65.40 ± 5.21	57.20 ± 6.74	56.10 ± 6.62
IEMOCAP	72.20 ± 3.14	76.60 ± 3.36	71.30 ± 4.31	69.28 ± 4.86
RAVDESS	73.50 ± 3.48	69.21 ± 4.69	65.28 ± 4.24	61.53 ± 4.73

**Table 5:** Comparison of speaker-dependent experiments with state-of-the-art approaches.

DATASET	Reference	Features	Accuracy (%)
**Emo-DB**	[59]	openSMILE features	84.62
	[60]	MFCCs, spectral centroids and MFCC derivatives	92.45
	[40]	Amplitude spectrogram and phase information	91.78
	[46]	3-D ACRNN	82.82
	[49]	ADRNN	90.78
	**Proposed**	**DCNN + CFS + SVM**	**95.10**
**SAVEE**	[59]	openSMILE features	72.39
	**Proposed**	**DCNN + CFS + SVM**	**82.10**
**IEMOCAP**	[35]	Convolution-LSTM	68
	[39]	Attention-BLSTM	64
	[45]	CNN + LSTM	64.50
	[46]	3-D ACRNN	64.74
	[49]	ADRNN	74.96
	**Proposed**	**DCNN + CFS + MLP**	**83.80**
**RAVDESS**	[60]	MFCCs, spectral centroids and MFCC derivatives	75.69
	[36]	Spectrogram + GResNet	64.48
	**Proposed**	**DCNN + CFS + SVM**	**81.30**

**Table 6:** Comparison of speaker-independent experiments with state-of-the-art approaches.

DATASET	Reference	Features	Accuracy (%)
**Emo-DB**	[31]	LLDs Stats	82.40
	[61]	Emobase feature set	76.90
	[37]	OpenSmile features + ADAN	83.74
	[47]	RESNET MODEL + Deep BiLSTM	85.57
	[48]	Complementary Features + KELM	84.49
	[49]	ADRNN	85.39
	[52]	DCNN + DTPM	87.31
	**Proposed**	**DCNN + CFS + MLP**	**90.50**
**SAVEE**	[31]	LLDs Stats	51.50
	[61]	eGeMAPs feature set	42.40
	**Proposed**	**DCNN + CFS + MLP**	**66.90**
**IEMOCAP**	[37]	OpenSmile features + ADAN	65.01
	[38]	IS10 + DBN	60.9
	[44]	SP + CNN	64.80
	[47]	RESNET MODEL + Deep BiLSTM	72.2
	[48]	Complementary Features + KELM	57.10
	[49]	ADRNN	69.32
	**Proposed**	**DCNN + CFS + SVM**	**76.60**
**RAVDESS**	**Proposed**	**DCNN + CFS + MLP**	**73.50**
